# A Combination of Metabolites Predicts Adherence to the Mediterranean Diet Pattern and Its Associations with Insulin Sensitivity and Lipid Homeostasis in the General Population: The Fenland Study, United Kingdom

**DOI:** 10.1093/jn/nxz263

**Published:** 2019-10-26

**Authors:** Tammy Y N Tong, Albert Koulman, Julian L Griffin, Nicholas J Wareham, Nita G Forouhi, Fumiaki Imamura

**Affiliations:** 1 MRC Epidemiology Unit, Institute of Metabolic Science, University of Cambridge School of Clinical Medicine, Cambridge, United Kingdom; 2 Cancer Epidemiology Unit, Nuffield Department of Population Health, University of Oxford, Oxford, United Kingdom; 3 National Institute for Health Research Biomedical Research Centres Core Nutritional Biomarker Laboratory, University of Cambridge, Addenbrooke's Hospital, Cambridge, United Kingdom; 4 National Institute for Health Research Biomedical Research Centres Core Metabolomics and Lipidomics Laboratory, University of Cambridge, Addenbrooke's Hospital, Cambridge, United Kingdom; 5 MRC Elsie Widdowson Laboratory, Cambridge, United Kingdom; 6 Department of Biochemistry, University of Cambridge, Cambridge, United Kingdom

**Keywords:** Mediterranean diet, molecular epidemiology, nutritional epidemiology, biomarkers, acylcarnitines, amines, sphingolipids, phospholipids, metabolomics, dietary pattern

## Abstract

**Background:**

Cardiometabolic benefits of the Mediterranean diet have been recognized, but underlying mechanisms are not fully understood.

**Objectives:**

We aimed to investigate how the Mediterranean diet could influence circulating metabolites and how the metabolites could mediate the associations of the diet with cardiometabolic risk factors.

**Methods:**

Among 10,806 participants (58.9% women, mean age = 48.4 y) in the Fenland Study (2004–2015) in the United Kingdom, we assessed dietary consumption with FFQs and conducted a targeted metabolomics assay for 175 plasma metabolites (acylcarnitines, amines, sphingolipids, and phospholipids). We examined cross-sectional associations of the Mediterranean diet score (MDS) and its major components with each metabolite, modeling multivariable-adjusted linear regression. We used the regression estimates to summarize metabolites associated with the MDS into a metabolite score as a marker of the diet. Subsequently, we assessed how much metabolite subclasses and the metabolite score would mediate the associations of the MDS with circulating lipids, homeostasis model assessment of insulin resistance (HOMA-IR), and other metabolic factors by comparing regression estimates upon adjustment for the metabolites.

**Results:**

Sixty-six metabolites were significantly associated with the MDS (*P* ≤ 0.003, corrected for false discovery rate) (Spearman correlations, *r*: −0.28 to +0.28). The metabolite score was moderately correlated with the MDS (*r* = 0.43). Of MDS components, consumption of nuts, cereals, and meats contributed to variations in acylcarnitines; fruits, to amino acids and amines; and fish, to phospholipids. The metabolite score was estimated to explain 37.2% of the inverse association of the MDS with HOMA-IR (*P* for mediation < 0.05). The associations of the MDS with cardiometabolic factors were estimated to be mediated by acylcarnitines, sphingolipids, and phospholipids.

**Conclusions:**

Multiple metabolites relate to the Mediterranean diet in a healthy general British population and highlight the potential to identify a set of biomarkers for an overall diet. The associations may involve pathways of phospholipid metabolism, carnitine metabolism, and development of insulin resistance and dyslipidemia.

## Introduction

The Mediterranean diet is a healthy dietary pattern associated with lower risk of cardiometabolic and other noncommunicable diseases (1–3). Despite the strong evidence for the health benefit from observational studies and controlled trials, the mechanisms through which the diet is associated with these disease outcomes are not well understood. Because the dietary pattern is characterized by diverse dietary factors including high consumption of vegetables, legumes, and fish; moderate consumption of fruits, cereals, nuts, eggs, dairy, white meat, and wine; and low consumption of red meat, processed meat, potato, and sweets (4), the diet is likely to influence disease outcomes via diverse pathways related to the different dietary factors (5–8).

The pathophysiology of noncommunicable diseases such as cardiovascular diseases, type 2 diabetes, or cancer may involve processes of chronic metabolite imbalance (9). Many small molecules in pathophysiologic processes can be profiled with metabolomics assays in blood, urine, and other tissues according to recent technological advancement (10, 11). Studies in the last few years, for example, have identified potential metabolomic biomarkers for type 2 diabetes, related to mechanisms of amino acids and lipid metabolism (10, 12). The metabolites are also influenced by environmental determinants including diet (13, 14). Research on diet, metabolites, and disease risk assessed simultaneously is promising to enhance understanding of how single dietary components or adherence to an overall dietary pattern influence disease risk (15). In addition, metabolomics may help identify objective markers of dietary intakes of foods or dietary patterns, or at least supplement traditional self-reported methods, because self-reported measurement of dietary exposure tends to involve misclassification (16). Single biomarkers (e.g., vitamins, fatty acids) also may not capture a complex dietary exposure (17, 18), and previous evidence showed the utility of a combination of biomarkers in capturing different types of food intake within a diet (19).

The effects of the Mediterranean diet on individual metabolite concentrations were evaluated in 2 studies in Spain (20–23): in substudies of the Prevencíon con Dieta Mediterránea (PREDIMED) trial, which included adults with high risk of cardiovascular diseases; and in the Metabolic Syndrome Reduction in Navarra (RESMENA) trial, which recruited individuals with a high mean BMI (36.1 kg/m^2^). No study to our knowledge has yet assessed how metabolites in combination would mediate the association of the Mediterranean diet with cardiometabolic risk factors in a general population. We conducted the current study evaluating targeted metabolomics data from 10,806 adults without diabetes in the Fenland Study in the United Kingdom, with the hypotheses that *1*) adherence to the Mediterranean diet would be associated with plasma metabolites and cardiometabolic risk factors (blood pressure, lipid concentrations, measures of insulin resistance, and glucose concentrations); and *2*) the association of adherence to the Mediterranean diet with cardiometabolic risk factors would be at least partly explained by a set of metabolites. We further examined whether the set of metabolites as a composite biomarker score was correlated with adherence to the Mediterranean diet.

## Methods

### Study population

The Fenland Study is a general population cohort which recruited 12,435 participants via general practices from 3 centers (Cambridge, Ely, Wisbech) in Cambridgeshire, United Kingdom (12, 24). Recruitment occurred between 2005 and 2015, and participants were eligible if they were born between 1950 and 1975. To investigate adults at risk of developing diabetes, adults with known diabetes were not considered eligible. Other exclusion criteria included pregnancy, an inability to walk unaided, psychosis, or terminal illness. All participants attended a single clinic visit during which they completed an FFQ and a general health and lifestyle questionnaire, and had blood samples and anthropometric measures taken by trained staff. The study was approved by the Health Research Authority Committee East of England—Cambridge Central and all participants gave written informed consent.

### Dietary assessment

Dietary assessment was by a 130-item semiquantitative FFQ which assessed dietary intake over the past year. The FFQ was previously examined for validity against 16-d weighed records, 24-h recall, and selected biomarkers (25, 26). There were 9 possible categories of intake frequencies, ranging from “never or less than once per month” to “6+ per day” for a standard food portion size. The reported frequency of intakes were converted into nutrients and dietary intakes as previously described (27). After dietary intakes were adjusted to a 2000-kcal/d (8.37-MJ/d) diet using the residual method, we calculated the Mediterranean diet score (MDS) representing the adherence to the Mediterranean dietary pyramid as previously described (2, 4). Briefly, we derived 15 dietary components (vegetables, legumes, fruits, nuts, cereals, dairy, fish, red meat, processed meat, white meat, egg, potato, sweets, alcohol, and olive oil) from the Mediterranean dietary pyramid and assigned continuous scores from 0 to 1 for each component (possible range: 0–15) according to the participant's level of adherence to the recommendation (**Supplemental Table 1**). Content validity of the MDS was confirmed with the inverse association with incident cardiovascular diseases and all-cause mortality in our previous work (2).

### Targeted metabolomics

Participants were instructed to fast for 10 h before their appointment time for the collection of fasting blood samples upon arrival, from which plasma samples were divided into aliquots. Using LC electrospray ionization and flow-injection analysis tandem MS, targeted metabolomics for 188 metabolites from the fasting plasma was performed at the Medical Research Council Human Nutrition Research, Cambridge, United Kingdom, using the Biocrates AbsoluteIDQ^®^ p180 kit (28, 29) as reported in our prior publication (12) and in the **Supplemental Text**. Briefly, samples were derivatized and extracted using a Hamilton STAR liquid handling station (Hamilton Robotics Ltd) and analyzed using a Waters-Acquity ultra-performance LC (Waters Ltd) system coupled to an ABSciex 5500 Qtrap mass spectrometer (Sciex Ltd). Data were processed in the Biocrates MetIDQ software. Metabolites included acylcarnitines, amino acids, biogenic amines, hexoses, phospholipids, and sphingolipids (12). Routine quality controls were performed in comparison to other laboratories using the same approach as described previously and in the Supplemental Text (30).

Of the 192 metabolites captured by the assay, 17 were excluded based on a prespecified exclusion criterion of >5% of participants showing peaks lower than the limit of quantification, because these measures were considered invalid [histamine, asymmetric dimethylarginine, carnosine, dihydroxyphenylalanine, dopamine, nitro-tyrosine, putrescine, spermine, 3 acylcarnitines (OH-C4, OH-C5, OH-C6, and C6), phosphatidylcholine diacyl 30:2, and 4 sphingomyelins (OH-24:1, 22:3, 26:0, and 26:1)]. For the current analyses, we evaluated 175 metabolites, including 40 acylcarnitines or carnitine, 35 amino acids or biogenic amines, total hexoses, 88 glycerophospholipids (14 acylated lysophosphatidylcholines, 74 phosphatidylcholines), and 11 sphingomyelins.

### Assessment of cardiovascular disease risk factors and other covariates

At the clinic visit, participants completed a health and lifestyle questionnaire about socioeconomic status, medication use, family history of diabetes, and smoking behavior. Weight (TANITA model BC-418 MA, Tanita), height (SECA 240, Seca), waist circumference (D loop tape, Chasmors Ltd), and blood pressure (Omron M4-1 automatic blood pressure monitor, Omrom Healthcare Inc.) were measured objectively (31). BMI was calculated as kg/m^2^. Physical activity was measured objectively using a combined heart rate monitor and movement sensor (Actiheart, CamNTech) which participants wore continuously for 6 d and 6 nights (32). Insulin, glucose, and blood lipids were measured using a fasting blood sample (Supplemental Text). Concentrations of LDL cholesterol were estimated with the Friedewald formula (33). HOMA-IR was calculated as insulin (milliunits per liter) times glucose (millimoles per liter) divided by 22.5 (34). Two-hour postload glucose (2-h glucose) was obtained after a standard oral-glucose-tolerance test using a 75-g glucose drink.

### Statistical analyses

All analyses were performed using Stata version 14.1 (Stata Corp). Unless corrected for multiple testing, α was 0.05. Missing covariates were imputed with multiple imputation (10 imputed data sets) (35), and the extent of missing information was <5.5% for all covariates. All analyses were based on 1 of the 10 imputed data sets, after confirming little variance across data sets. Missing information on metabolites existed because of exclusion of a few batches, and therefore analyses for different metabolites varied in sample sizes. Undetectable peaks of metabolites were replaced with a random value between the batch-specific minimum value and 0.1 times the batch-specific minimum value. To minimize the influence of outliers, the variables were winsorized using cutoffs based on the batch-specific mean plus or minus 5 times the batch-specific SD. Batch correction was then applied to control for variability (batch-specific means and SDs) across laboratory batches (27).

All the 175 metabolites were log transformed to improve normality of distribution and to allow interpretation of relative differences in each metabolite per SD difference in the MDS. Linear regressions were fitted to model each of the 175 plasma metabolites as a dependent variable and standardized MDS as an independent variable. Analyses were adjusted for potential confounders including age, sex, test site, education level, occupation, income, smoking, physical activity, energy expenditure, medication use, family history of diabetes, BMI, and waist circumference. Significant associations were then identified, based on the α corrected for the false discovery rate (36). Considering that anthropometric measures may be mediators or outcomes of diet–metabolite associations, we repeated the analyses for the MDS and the metabolites without adjustment for anthropometric measures (BMI and waist circumference).

To assess whether the metabolites collectively represented adherence to the overall Mediterranean diet, a metabolite score was generated based on the strengths of associations of metabolites with the MDS. Briefly, *1*) we selected the metabolites significantly associated with the MDS in the total sample; *2*) we fitted a multivariable-adjusted model to predict the MDS simultaneously by the selected metabolites using a randomly selected half of the study sample (derivation set); *3*) the regression coefficient for each of the selected metabolites was then multiplied as a weight with each metabolite in the second half of the data set (test set); *4*) the products were summed to generate a metabolite score in the test set; and *5*) the score was standardized to have mean = 0 and SD = 1. The same steps (1–5) were repeated by flipping the derivation and test sets, so that a metabolite score for each participant was derived from an independent sample, i.e., avoiding the problem of “over-optimism” (37, 38).

Additional analyses were performed to confirm the utility and robustness of the metabolite score. As a secondary analysis, plasma vitamin C was examined as a biomarker for its correlation (Spearman's ρ) with the MDS and the metabolite score to confirm whether the set of metabolites performed better than a single biomarker of diet quality in capturing adherence to the Mediterranean diet. To assess influences of metabolite selection concerning collinearity between metabolites, we repeated the process and derived additional metabolite scores using metabolites selected from a multivariable-adjusted backward stepwise regression to predict the MDS (cutoff of *P* values = 0.01 for entry and 0.05 for removal). To examine internal reproducibility of the metabolite score within the Fenland cohort, we defined a derivation set and a test set by age, sex, BMI, smoking status, and study sites (Cambridge, Ely, and Wisbech) separately. In addition, to examine how each individual dietary component contributed to the association of the Mediterranean diet with metabolite concentrations, linear regressions of the MDS with metabolites were repeated sequentially adjusting for each dietary component.

### Analyses to assess influences of metabolites on associations of the MDS with cardiometabolic risk factors

We assessed the influence of metabolites on the association between the Mediterranean diet and cardiometabolic risk factors: systolic blood pressure (SBP), diastolic blood pressure (DBP), HDL cholesterol, LDL cholesterol, ratio of total cholesterol to HDL cholesterol, triglycerides (log transformed), HOMA-IR, and 2-h glucose. We evaluated all metabolites significantly associated with the Mediterranean diet by using variables of the metabolite score, the metabolite subclasses, and individual metabolites. To assess the degree of influence of the metabolites, the percentage change of regression coefficients of models before and after addition of metabolites was calculated as 100 × [(β_0_ − β_1_)/β_0_], where β_0_ and β_1_ represent the association of the Mediterranean diet with each cardiometabolic risk factor before and after adjustment for metabolites assessed as potential mediators (39, 40). We considered the percentage of attenuation of the association between the Mediterranean diet and cardiometabolic risk factors as the degree of mediation due to the metabolites. SEs and CIs for the percentage attenuation were estimated by bootstrapping with 2000 iterations (39). *P* values were based on a Wald test for an estimate of the percentage divided by the bootstrap SE (α = 0.05).

## Results

### Baseline characteristics

Baseline characteristics of the Fenland participants are reported in **Table 1**, by thirds of adherence to the MDS. On average, participants who had higher (tertile 3) adherence to the Mediterranean diet were more likely to be women or nonsmokers, to report no family history of diabetes, and to have higher socioeconomic status. They also tended to have lower BMI, waist circumference, HOMA-IR, blood pressure, LDL cholesterol, and ratio of total cholesterol to HDL cholesterol.

**TABLE 1:** Baseline characteristics of participants by thirds of adherence to the Mediterranean diet: the Fenland Study, 2005–2015^1^

	Adherence to the Mediterranean diet		
Baseline characteristics	Tertile 1 (3.3–8.4 points) (*n* = 3657)	Tertile 2 (8.4–9.7 points) (*n* = 3615)	Tertile 3 (9.7–14.0 points) (*n* = 3534)
Age, y	48.2 ± 7.3	48.4 ± 7.5	48.3 ± 7.6
Test site			
Cambridge	24.3	34.0	47.8
Ely	37.3	38.8	34.6
Wisbech	38.4	27.2	17.6
Sex, women	40.3	54.9	65.5
Education level^2^			
Compulsory	28.8	20.1	12.0
Further	52.4	46.7	39.1
Higher	18.8	33.2	48.9
Occupation^3^			
Routine/tech/others	55.4	40.2	29.1
Managerial/professional	44.6	59.8	70.9
Household income			
≤£25,000	43.7	32.6	26.3
£25,001–44,775	31.2	35.7	34.7
≥£44,776	25.1	31.7	39.0
Smoking			
Never	52.6	54.4	56.0
Former	28.5	35.0	36.0
Current	18.9	10.6	8.0
Energy expenditure, kJ · kg^–1^ · d^–1^	55.2 ± 23.4	52.6 ± 21.4	54.1 ± 21.3
Medication use	42.6	43.4	40.6
Family history of diabetes	20.2	22.2	19.0
BMI, kg/m^2^	27.7 ± 4.8	27.0 ± 4.9	25.9 ± 4.5
Waist circumference, cm	94.5 ± 13.4	90.9 ± 13.5	87.1 ± 12.8
Fasting insulin, pmol/L	53.9 ± 40.0	47.3 ± 31.7	41.6 ± 37.5
Fasting glucose, mmol/L	4.9 ± 0.8	4.8 ± 0.6	4.8 ± 0.6
2-h glucose, mmol/L	5.4 ± 1.8	5.3 ± 1.7	5.1 ± 1.6
HOMA-IR	12.3 ± 10.9	10.6 ± 8.2	9.2 ± 9.5
Systolic blood pressure, mm Hg	125.1 ± 15.3	122.9 ± 15.1	120.4 ± 15.2
Diastolic blood pressure, mm Hg	76.2 ± 10.3	74.6 ± 10.0	72.9 ± 9.9
Total cholesterol, mmol/L	5.5 ± 1.0	5.4 ± 1.0	5.4 ± 1.0
HDL-C, mmol/L	1.4 ± 0.4	1.5 ± 0.4	1.6 ± 0.4
LDL-C, mmol/L	3.5 ± 0.9	3.4 ± 0.9	3.3 ± 0.9
Cholesterol:HDL-C ratio	4.1 ± 1.3	3.8 ± 1.2	3.5 ± 1.1
Triglycerides, mmol/L	1.3 ± 0.9	1.2 ± 0.8	1.0 ± 0.7
Energy intake, kcal/d	2123.2 ± 739.5	1925.0 ± 655.8	1860.7 ± 603.6

1 Total *n* = 10,806. Values are means ± SDs or percentages. Adherence to the Mediterranean diet among 10,806 participants recruited at baseline in 2005–2015 in the Fenland Study, using the dietary score derived from the Mediterranean dietary pyramid (see the Methods for details; possible range: 0–15). HDL-C, HDL cholesterol; LDL-C, LDL cholesterol.

2 For education, compulsory included “school leaving certificate,” “CSE,” and “GCE O level or GCSE”; further included “matriculation,” “GCE A level, AS level, highers,” “technical college exams, city & guilds,” “HND GNVQ,” “completed apprenticeship,” “secretarial college exams,” “teaching diploma, HNC, NVQ,” and “trade certificates”; higher included “university degree.”

3 For occupation, routine/tech/others included clerical, technical, semiroutine, and routine jobs; managerial/professional included modern professional, senior manager, middle management, and traditional professional jobs.

### Association of the Mediterranean diet with metabolite concentrations

**FIGURE 1:**
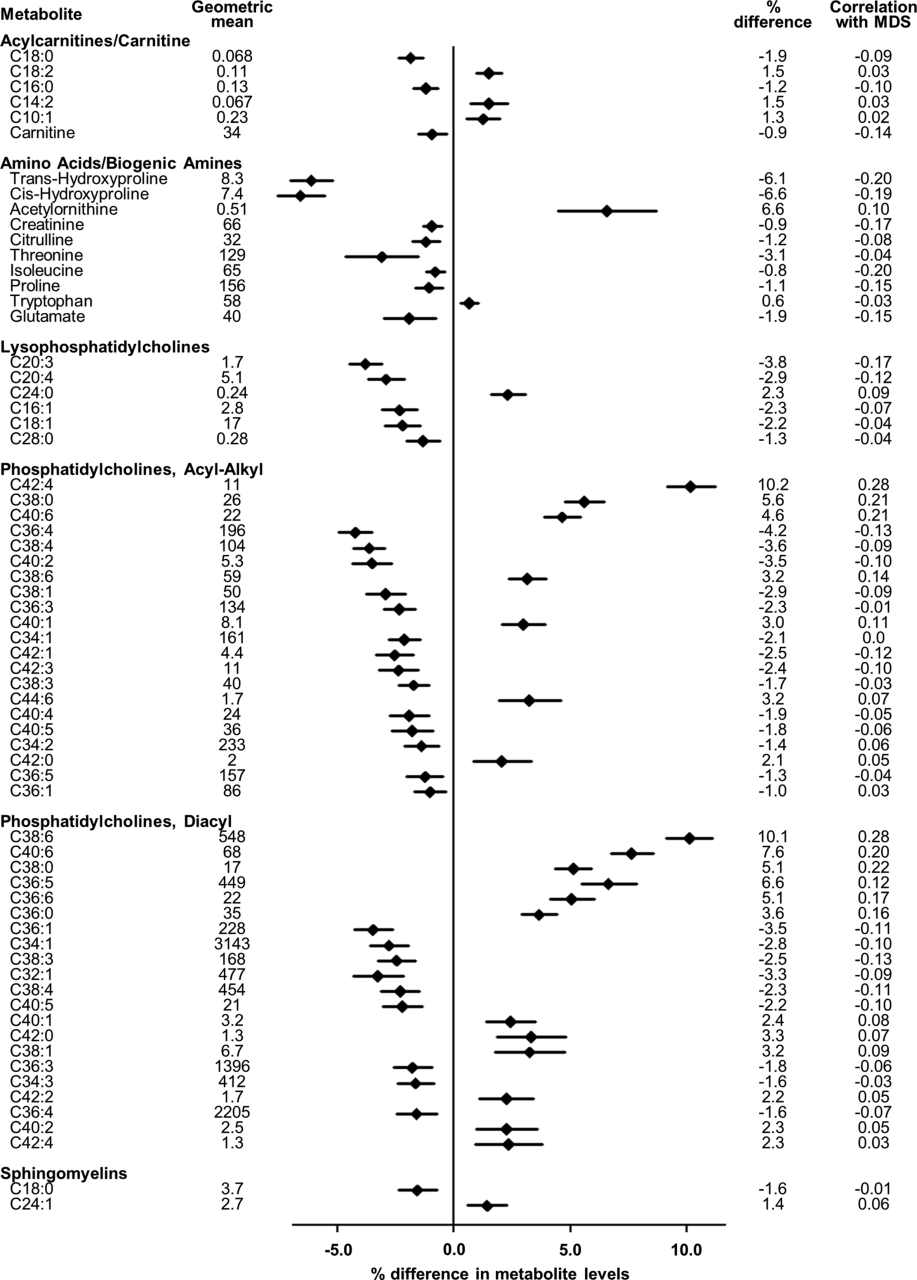
Association of the Mediterranean diet with metabolite concentrations: the Fenland Study (*n* = 10,806, 2005–2015). Percentage difference in metabolite concentrations based on linear regression fitted to data from 10,806 participants (recruited in 2005–2015) for an association of the MDS (per SD) with each of the log-transformed metabolites. Geometric means of metabolites measured in micromoles per liter for the amino acids and biogenic amines, and relative concentrations for the other metabolites. For acylcarnitines, phospholipids, and sphingolipids, numbers of carbons and double bonds of acyl moieties are presented. Correlation represents Spearman's ρ with the MDS. MDS, Mediterranean diet score.

Among 10,806 adults in total (*n* = 7338–10,725 for individual metabolites), 66 metabolites were significantly associated with the MDS, after correction for the false discovery rate (*P* ≤ 0.003) (**Figure 1**). These included 6 acylcarnitines or carnitine, 10 amino acids or biogenic amines, 48 phospholipids (6 acylated lysophosphatidylcholines, 21 acyl-alkyl phosphatidylcholines, 21 diacyl phosphatidylcholines), and 2 sphingomyelins. Directions of the associations were heterogeneous within each class of metabolite. For example, unsaturated acylcarnitines (18:2, 14:2, and 10:1) were positively associated, whereas saturated (16:0, 18:0) or free carnitines (0) were inversely associated. Of essential amino acids, tryptophan was positively associated, whereas isoleucine (one of the branched-chain amino acids) and threonine were negatively associated. Leucine and valine (the other 2 branched-chain amino acids) were significantly negatively associated with the MDS before adjustment for BMI and waist circumference, but the associations were attenuated toward the null and became nonsignificant after adjustment. Of the 66 metabolites significantly associated with the MDS, 5 metabolites (acylated lysophosphatidylcholine 18:1, acyl-alkyl phosphatidylcholines 34:2, 36:5, 36:1, and tryptophan) were nonsignificant in the model unadjusted for adiposity (BMI and waist circumference).

When these 66 metabolites were subsequently summarized as a metabolite score, this had a moderate correlation with the MDS (ρ = 0.43) (**Table 2**). For comparison, the correlation between the MDS and plasma vitamin C was 0.26. Correlations of similar magnitude remained when the metabolite score was summarized based on metabolites selected from backwards stepwise regression or when the derivation set and test set were defined by strata of age, sex, BMI, smoking status, or study site (ρ = 0.42–0.43) (Table 2).

**TABLE 2:** Correlations between the MDS and metabolite scores in the derivation and test sets: the Fenland Study, 2005–2015^1^

Derivation set			Test set^2^		
Strata	*n*	*ρ* ^3^	Strata	*n*	*ρ*
Metabolite score					
Random half 1	5382	0.47	Random half 2	5424	0.43
Random half 2	5424	0.45	Random half 1	5382	0.44
			Pooled^4^		0.43
Metabolite score from backwards regression					
Random half 1	5382	0.45	Random half 2	5424	0.42
Random half 2	5424	0.43	Random half 1	5382	0.42
			Pooled		0.42
Metabolite score stratified by age, y					
≤48.5	5424	0.48	>48.5	5382	0.42
>48.5	5382	0.46	≤48.5	5424	0.43
			Pooled		0.42
Metabolite score stratified by sex					
Men	5031	0.46	Women	5775	0.40
Women	5775	0.43	Men	5031	0.43
			Pooled		0.43
Metabolite score stratified by BMI, kg/m^2^					
≤26.2	5470	0.47	>26.2	5336	0.37
>26.2	5336	0.40	≤26.2	5470	0.43
			Pooled		0.43
Metabolite score stratified by smoking status					
Former or current	4934	0.50	Never	5874	0.42
Never or current	7236	0.46	Former	3572	0.42
Never or former	9446	0.44	Current	1362	0.40
			Pooled		0.43
Metabolite score stratified by study site^5^					
Cambridge and Ely	7794	0.46	Wisbech	3012	0.38
Cambridge and Wisbech	6817	0.48	Ely	3989	0.40
Ely and Wisbech	7001	0.43	Cambridge	3805	0.41
			Pooled		0.42

1 Total *n* = 10,806. MDS, Mediterranean diet score.

2 Scoring weights were derived from a derivation set, then applied to independent samples (a test set) within the Fenland Study. Then, the mass Spearman's ρ between the metabolite score and the MDS was calculated in the test set.

3 *ρ* represents Spearman's ρ of the metabolite scores with the MDS based on the Mediterranean dietary pyramid within the derivation set (subject to an overfitting problem).

4 Spearman's ρ between the metabolite score and the MDS was calculated by pooling 2 or 3 independent test sets.

5 Three study sites of the Fenland Study (Cambridge, Ely, and Wisbech in Cambridgeshire, United Kingdom).

Dietary components of the Mediterranean diet were evaluated to assess their contributions to the association of the Mediterranean diet with metabolites (**Supplemental Figure 1**). The associations between the MDS and acylcarnitines were attenuated by adjusting for nuts, cereals, red meats, or processed meats, suggesting that these dietary components mediated the observed associations between the MDS and acylcarnitines. Similarly, fruits attenuated the associations of the MDS with amino acids and biogenic amines; and fish attenuated the association of the MDS with phospholipids. No single dietary component explained the associations of the MDS with all the metabolites consistently, and no metabolite was associated with all food groups included in the MDS. Olive oil and dairy products did not appreciably contribute to any of the associations of the MDS with the metabolites.

### Potential influences of metabolites on associations of the MDS with cardiometabolic risk factors

After adjusting for potential confounders and mediators, higher MDS was associated with higher concentrations of HDL cholesterol, a lower ratio of cholesterol to HDL cholesterol, lower triglycerides, and lower HOMA-IR (**Table 3**), but not significantly associated with SBP, DBP, LDL cholesterol, and 2-h glucose (**Supplemental Table 2**). When we added groups of metabolites to the regression models, significant attenuation in the association was observed for the ratio of cholesterol to HDL cholesterol, triglycerides, and HOMA-IR (Table 3). For example, the association between the MDS and HOMA-IR was significantly attenuated by adjustment for acylcarnitines and carnitine (11.3% attenuation), amino acids and biogenic amines (26.4%), acyl-alkyl phosphatidylcholines (20.8%), diacyl phosphatidylcholines (27.0%), and sphingolipids (8.4%). The addition of the metabolite score significantly attenuated the association between the MDS and HOMA-IR (37.2%), but not the other outcomes.

**TABLE 3:** Contribution of groups of metabolites to the association between the Mediterranean diet and cardiovascular disease risk factors: the Fenland Study, 2005–2015^1^

	HDL-C, mmol/L		Cholesterol:HDL-C ratio		Triglycerides, %		HOMA-IR, %	
	Difference (95% CI)^2^	% Attenuation^3^	Difference (95% CI)^2^	% Attenuation^3^	Difference (95% CI)^2^	% Attenuation^3^	Difference (95% CI)^2^	% Attenuation^3^
Reference model^3^	0.02 (0.01, 0.02)		−0.05 (−0.07, −0.02)		−1.99 (−2.99, −0.99)		−3.42 (−4.43, −2.40)	
Adjusted for metabolite subclasses								
+ acylcarnitines	0.02 (0.01, 0.02)	−2.0	−0.03 (−0.05, −0.00)	44.7**	−1.01 (−1.99, −0.01)	48.3**	−2.99 (−4.03, −1.94)	11.3***
+ amino acids/biogenic amines	0.01 (0.01, 0.02)	17.0	−0.05 (−0.07, −0.02)	1.9	−1.98 (−3.03, −0.91)	−4.6	−2.21 (−3.30, −1.11)	26.4**
+ lysophosphatidylcholines	0.02 (0.01, 0.03)	−12.6	−0.03 (−0.06, −0.01)	26.0	−0.63 (−1.58, 0.32)	67.1*	−3.34 (−4.40, −2.39)	−1.1
+ phosphatidylcholine acyl-alkyls	0.01 (0.01, 0.02)	8.2	−0.04 (−0.07, −0.02)	7.3	−1.81 (−2.74, −0.87)	2.3	−2.52 (−3.64, −1.39)	27.0***
+ phosphatidylcholine diacyls	0.02 (0.01, 0.02)	−1.7	−0.06 (−0.08, −0.03)	−4.3	−1.40 (−2.37, −0.43)	37.8*	−2.68 (−3.82, −1.53)	20.8**
+ sphingolipids	0.02 (0.01, 0.02)	7.7	−0.03 (−0.05, −0.01)	31.1**	−1.32 (−2.32, −0.29)	32.0	−3.02 (−4.04, −1.98)	8.4**
Adjusted for the metabolite score								
+ metabolite score^4^	0.01 (0.00, 0.02)	20.6	−0.03 (−0.06, −0.01)	35.6	−2.01 (−3.37, −0.63)	8.9	−1.76 (−3.12, −0.37)	37.2*

1 Total *n* = 10,806. *P < 0.05, **P < 0.01, ***P < 0.001 for percentage changes. HDL-C, HDL cholesterol.

2 Values represent unit or percentage differences in cardiovascular disease risk factors [β coefficients or exp(β) and corresponding 95% CIs]; and percentage change in β (see footnote 3). Regression models were fitted with adjustment for age, sex, test site, education level, income, occupation, medication use, family history of diabetes, objectively measured physical activity, smoking, BMI, and waist circumference. Adherence to the Mediterranean diet was not significantly associated with systolic blood pressure, diastolic blood pressure, and 2-h glucose (*P* > 0.05) and therefore these phenotypes were not considered as potential mediation (see results in Supplemental Table 2).

3 Percentage changes in β coefficients were calculated as changes in β coefficients from those of the “reference model” upon statistical adjustment for the metabolite score or metabolite subclasses (mediation analysis). As availability of metabolites varied, sample sizes were different across models: reference model, *n* = 10,806; + acylcarnitines, *n* = 10,701; + amino acids/biogenic amines, *n* = 9224; + lysophosphatidylcholines, *n* = 10,718; + phosphatidylcholine acyl-alkyls, *n* = 9868; + phosphatidylcholine diacyls, *n* = 8946; + sphingomyelins, *n* = 10,690; + metabolite score, *n* = 7138.

4 Metabolite score included all 66 metabolites associated with the Mediterranean diet derived in a random half of the total data set and validated in the second half, weighted by their respective regression coefficients.

**FIGURE 2:**
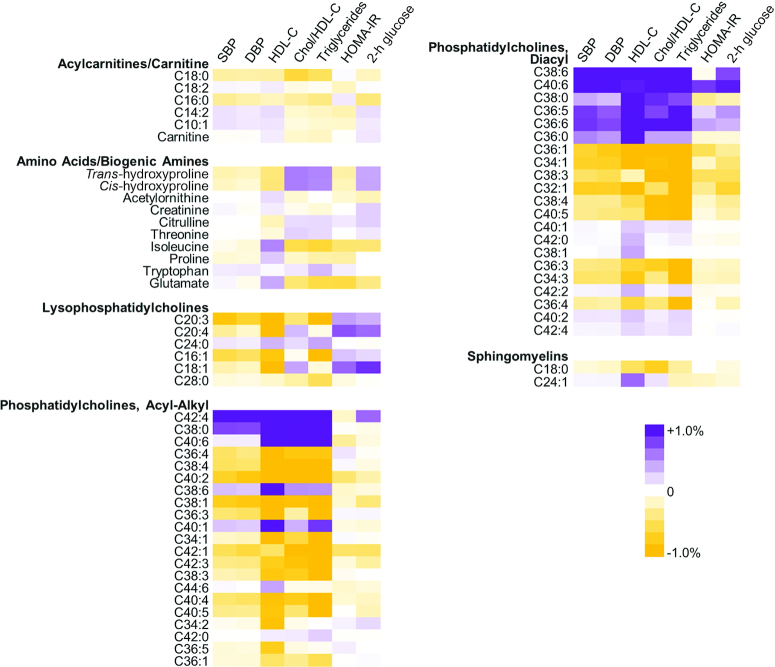
Contribution of metabolites to the association between the Mediterranean diet and cardiovascular disease risk factors: the Fenland Study (*n* = 10,806, 2005–2015). Purple indicates contribution of the particular metabolite to the association of the Mediterranean diet and 1 of the outcomes (the association was attenuated when adjusted for the metabolite). Orange indicates no contribution, where adjustment for the metabolite strengthened the association. All estimates were adjusted for age, sex, test site, education level, income, occupation, medication use, family history of diabetes, objectively measured physical activity, smoking, BMI, and waist circumference. For acylcarnitines, phospholipids, and sphingolipids, numbers of carbons and double bonds of acyl moieties are presented. Chol/HDL-C, cholesterol:HDL cholesterol ratio; DBP, diastolic blood pressure; HDL-C, HDL cholesterol; SBP, systolic blood pressure.

The influence of individual metabolites on cardiometabolic risk factors is shown in **Figure 2**. Overall, we observed that some individual acyl-alkyl phosphatidylcholines (e.g., 42:4, 38:0, 40:6) and diacyl phosphatidylcholines (e.g., 38:6, 40:6) contributed to explaining the association between the MDS and cardiometabolic risk factors (cholesterol:HDL cholesterol ratio, triglycerides, HOMA-IR, and 2-h glucose), as indicated by the attenuation in regression coefficients after adjustment for the metabolite.

## Discussion

In a population-based cohort of adults without diabetes in the United Kingdom, 66 metabolites across different subclasses were found to be associated with adherence to the Mediterranean diet. Among multiple dietary components, no single dietary factor explained the association of adherence to the Mediterranean diet with these metabolites. A set of metabolites such as carnitines, amines, and phospholipid species moderately explained the association of the Mediterranean diet with metabolic risk factors of insulin resistance and dyslipidemia. The results of the current study highlight the potential utility of combining multiple metabolites together as a composite marker of diet quality, and the potential application of both individual and multiple metabolites in diet–disease etiological research.

### Comparison with other studies

Two intervention trials in Spain (a subset of PREDIMED and RESMENA) reported the associations between the Mediterranean diet and metabolite concentrations (20–23). Neither study examined the variability of adherence to the Mediterranean diet that could be captured by a set of metabolites, or whether or to what extent the metabolites could explain associations of the Mediterranean diet with major cardiometabolic risk factors. Because both trials assessed changes in metabolite concentrations over the intervention period, the results may not be directly comparable with those from our cross-sectional study. In 1 of the 3 substudies in the PREDIMED trial (*n* = 980), the authors reported a higher risk of CVD in the top than in the bottom quartile of short- and medium-chain acylcarnitines in the control groups, but no significant effect of the intervention on plasma concentrations of short-, medium-, and long-chain acylcarnitines over 1 y of follow-up (21). No short-chain acylcarnitine was correlated with the MDS in our study, but we found that adherence to the Mediterranean diet was associated with 2 acylcarnitines with the same number of carbons (18:0 and 18:2) in opposing directions, which highlights the importance of examining degrees of saturation of fatty acyl chains in addition to chain length. In the other substudy of PREDIMED (*n* = 98), the authors reported that a Mediterranean diet intervention increased concentrations of urinary proline and urinary creatinine compared with a control low-fat diet, which was contrary to our findings based on plasma samples (20). The differences in the study populations and in biological samples of plasma or urine limit comparability between the observations in different studies and warrant further investigations on amines. In the third substudy from the PREDIMED, plasma lipidomics analysis was performed. The authors reported results consistent with our study for several phosphatidylcholines (36:4, 38:4, 38:3) and 1 sphingomyelin (24:1), but an opposite direction of association for 18:0 sphingomyelin (23). This inconsistency indicates sensitivity of specific circulating lipids to the difference between the British self-reported diet and the intervention, of providing nuts or olive oil as well as the Mediterranean diet advice.

Our findings were generally in agreement with the findings from the RESMENA study (*n* = 72), a 6-mo trial of a Mediterranean diet intervention (22). Both the RESMENA trial and our study identified significant associations of the Mediterranean diet with specific metabolites, including 3 acylated lysophosphatidylcholines (20:3, 20:4, and 16:1), acyl-alkyl phosphatidylcholine 38:6, and 9 diacyl-phosphatidylcholines (38:6, 36:1, 34:1, 38:3, 32:1, 38:4, 36:3, 34:3, and 36:4). In contrast, associations were inconsistent for acyl-alkyl phosphatidylcholine 38:4, lysophosphatidylcholine 18:1, and phosphatidylcholine 36:4. This inconsistency could reflect the differences in constituents of the Mediterranean diet and in population characteristics. For example, an increased concentration of lysophosphatidylcholine 18:1 in the RESMENA trial might reflect higher consumption of olive oil or other plant-based oils, whereas no significant association in our study may have partly reflected suppression of de novo synthesis of 18:1 due to low consumption of those plant oils on average. The inconsistency highlights the possible importance of specific vegetable oils; moreover, phospholipid subtypes require further investigation in different populations.

### Interpretation of findings and implications

Although many of the targeted metabolites are known to be synthesized de novo, we found that the combination of 66 metabolites had a moderate correlation of 0.43 with the MDS. In contrast, the correlation of plasma vitamin C, a marker of dietary intakes of vitamin C or food groups rich in vitamin C (19), with the MDS was 0.26. These findings suggest that our approach using metabolomics could identify a set of small metabolites as a biomarker of adherence to the Mediterranean diet or of overall diet quality. Further research is warranted to establish the utility of the metabolite score in different populations and to test its validity using dietary feeding studies.

The secondary analyses focusing on dietary components showed that, for example, the association of the MDS with acylcarnitines and carnitine attenuated in the regression model adjusted for nuts, cereals, red meats, or processed meats. Acylcarnitines are derivatives of carnitines from mitochondrial fatty acid oxidation, and meats are their dietary sources (15, 41). Thus, the findings for red and processed meats are plausible. By contrast, how nuts and cereals could lead to the attenuation of the observed associations is unclear, requiring further research on dietary sources and bioactive elements in nuts and cereals that could determine concentrations of acylcarnitines, which are related to poorer cardiovascular health (42, 43). This indicates that small metabolites reflect both dietary intakes and metabolic states altered by dietary exposure.

Our finding for fish consumption also indicates the potential impact of a single dietary factor on multiple metabolite species. Fish consumption appeared to explain the association of the Mediterranean diet with many phospholipids including phosphatidylcholines, which include lyso-, acyl-aklyl- and diacyl-phosphatidylcholines that are key components of cellular membranes and blood lipoproteins (15, 44). One of the dietary sources of choline is fish (15, 44), and thus our finding is plausible. Fish consumption also attenuated the inverse associations between adherence to the Mediterranean diet and carnitines with long-chain SFAs. This could potentially reflect that fish consumption, for example, supplied dietary PUFAs, thereby suppressing de novo fatty acid synthesis. As well as acyl-carnitines, subtypes of acyl-chains of phospholipids should be studied further.

Our findings from the mediation analysis indicate, under the assumption that the associations were causal, that higher adherence to the Mediterranean diet would improve insulin resistance and dyslipidemia and also alter the profile of many circulating metabolites including carnitines, amines, phospholipids, and sphingomyelins. Although causality cannot be determined in this study, these findings suggest that multiple metabolites are involved in the etiology of cardiometabolic diseases and also influenced by the overall dietary pattern.

### Strengths and limitations

To our knowledge, our study is the largest study to date examining the association between the Mediterranean diet and metabolite concentrations via a metabolomics approach. With its large sample size, internal cross-validation analysis confirmed the internal validity of the metabolite score. Limitations of our work included the cross-sectional design of the Fenland Study, limiting the interpretation of the causal pathways. For instance, metabolite concentrations could vary as a consequence of the causal effect of the diet on insulin resistance. Although we adjusted for major potential confounders, residual confounding could be present and adjustment for a metabolite in assessing mediation could cause further residual confounding (40). Nonetheless, it is still meaningful that a mechanism of causation, confounding, or both could indeed involve specific small molecules measured by metabolomics. Measurement errors in dietary measures, the metabolomics assays, and covariates could exist and cause both false-positive and false-negative findings for the associations of the MDS with metabolites.

Another limitation is that our targeted metabolomics assay did not include some molecules relevant to the Mediterranean diet, such as dietary flavonoids. We focused on relative differences in metabolite concentrations by adherence to the Mediterranean diet, although absolute concentrations would potentially be of interest. The assay platform we used also could not differentiate isobaric lipids with identical molecular weights. Acyl-chains of phosphatidylcholine 38:6, for example, could be both a pair of 16:0 and 22:6 (palmitic acid and DHA) and a pair of 18:1 and 20:5 (oleic acid and EPA). Degrees of both chain-length and -saturation influence roles of fat-esterified molecules. Therefore, roles of individual phospholipids without information on acyl-chains cannot be interpreted in this study and should be investigated in future work, for example, by using NMR spectroscopy or alternative MS platforms (45, 46). Collinearity between metabolites would be concerning but our cross-validation approach confirmed the robustness of the results derived from a combination of metabolites.

The generalizability of our findings is limited because this study recruited adults without diabetes and mainly adults of European descent (92%). Because the Mediterranean diet is likely to be fit to dietary habits uniquely in different populations in Mediterranean and non-Mediterranean countries (2, 47), future investigation in other populations is warranted. For example, adherence to the Mediterranean diet observed in our study was not necessarily the same as that in Mediterranean countries, regarding mean consumption of olive oil, nuts, and yogurt. Previous assessments of Mediterranean diet adherence across different regions have found that Northern European countries including the United Kingdom had lower Mediterranean diet adherence than the Mediterranean countries, but that adherence was also higher in the United Kingdom in the 2000–2003 period than in the 1961–1965 period (48). We also did not differentiate between certain details of Mediterranean diet adherence, for example, on red wine compared with alcohol consumption, because our dietary assessment did not differentiate between red wine and white wine. The difference could influence a combination of metabolites to predict adherence to the Mediterranean diet. Alongside the possibility that different components of the Mediterranean diet could contribute differentially between populations, the lack of replication of our findings in an independent cohort is one of the limitations of our study. Confirmatory work would ideally be conducted in both the United Kingdom and other countries.

### Conclusions

The current findings provide evidence that different subclasses of small metabolites including carnitines, amines, and phospholipids were associated with adherence to the Mediterranean diet, and should prompt the discovery of potential biomarkers or metabolic profiles associated with this dietary pattern. In addition, the results also showed that the associations of the Mediterranean diet with measures of insulin resistance and major lipid profiles were partly explained by metabolites related to the Mediterranean diet, suggesting their involvement in pathways linking diet to disease risk. Overall, these findings advance greater understanding of metabolites as potential dietary biomarkers and help with knowledge on pathways involved in diet–disease etiology.

## Supplementary Material

nxz263_Supplementary_Files

## Notes

Supported by Medical Research Council (MRC) Epidemiology Unit grants MC_UU_12015/5 and MC_UU_12015/1 (to TYNT, NJW, FI, and NGF), National Institute for Health Research (NIHR) Biomedical Research Centre grant IS-BRC-1215-20014 (to NGF, AK, and NJW), and MRC Elsie Widdowson Laboratory grant MC_UD99999906 (to AK and JLG). The Fenland Study was funded by the MRC (DOI: 10.1186/ISRCTN72077169). Metabolomics analyses were funded by MRC grant on Omic approaches MC_PC_13046 (to NJW). NJW is an NIHR senior investigator.

Author disclosures: The authors report no conflicts of interest.

Supplemental Tables 1 and 2, Supplemental Text, and Supplemental Figure 1 are available from the “Supplementary data” link in the online posting of the article and from the same link in the online table of contents at https://academic.oup.com/jn/.

NGF and FI contributed equally to this work.

Abbreviations used: DBP, diastolic blood pressure; MDS, Mediterranean diet score; PREDIMED, Prevencíon con Dieta Mediterránea; RESMENA, Metabolic Syndrome Reduction in Navarra; SBP, systolic blood pressure.

## References

[1] Estruch R, Ros E, Salas-Salvadó J, Covas M-I, Corella D, Arós F, Gómez-Gracia E, Ruiz-Gutiérrez V, Fiol M, Lapetra J et al. Primary prevention of cardiovascular disease with a Mediterranean diet. N Engl J Med. 2013;368:1279–90. 29897867 10.1056/NEJMc1806491

[2] Tong TYN, Wareham NJ, Khaw K-T, Imamura F, Forouhi NG Prospective association of the Mediterranean diet with cardiovascular disease incidence and mortality and its population impact in a non-Mediterranean population: the EPIC-Norfolk study. BMC Med. 2016;14:135. 27679997 10.1186/s12916-016-0677-4 PMC5041408

[3] Sofi F, Macchi C, Abbate R, Gensini GF, Casini A Mediterranean diet and health status: an updated meta-analysis and a proposal for a literature-based adherence score. Public Health Nutr. 2014;17:2769–82. 24476641 10.1017/S1368980013003169 PMC10282340

[4] Bach-Faig A, Berry EM, Lairon D, Reguant J, Trichopoulou A, Dernini S, Medina FX, Battino M, Belahsen R, Miranda G et al. Mediterranean diet pyramid today. Science and cultural updates. Public Health Nutr. 2011;14:2274–84. 22166184 10.1017/S1368980011002515

[5] Barclay AW, Petocz P, McMillan-Price J, Flood VM, Prvan T, Mitchell P, Brand-Miller JC Glycemic index, glycemic load, and chronic disease risk—a meta-analysis of observational studies. Am J Clin Nutr. 2008;87:627–37. 18326601 10.1093/ajcn/87.3.627

[6] Chowdhury R, Warnakula S, Kunutsor S, Crowe F, Ward HA, Johnson L, Franco OH, Butterworth AS, Forouhi NG, Thompson SG et al. Association of dietary, circulating, and supplement fatty acids with coronary risk: a systematic review and meta-analysis. Ann Intern Med. 2014;160:398–406. 24723079 10.7326/M13-1788

[7] López-Miranda J, Pérez-Jiménez F, Ros E, De Caterina R, Badimón L, Covas MI, Escrich E, Ordovás JM, Soriguer F, Abiá R et al. Olive oil and health: summary of the II International Conference on Olive Oil and Health Consensus Report, Jaén and Córdoba (Spain) 2008. Nutr Metab Cardiovasc Dis. 2010;20:284–94. 20303720 10.1016/j.numecd.2009.12.007

[8] Lippi G, Franchini M, Favaloro EJ, Targher G Moderate red wine consumption and cardiovascular disease risk: beyond the ‘French paradox’. Semin Thromb Hemost. 2010;36:59–70. 20391297 10.1055/s-0030-1248725

[9] Whitfield PD, German AJ, Noble P-JM Metabolomics: an emerging post-genomic tool for nutrition. Br J Nutr. 2004;92:549–55. 15522124 10.1079/bjn20041243

[10] Roberts LD, Koulman A, Griffin JL Towards metabolic biomarkers of insulin resistance and type 2 diabetes: progress from the metabolome. Lancet Diabetes Endocrinol. 2014;2:65–75. 24622670 10.1016/S2213-8587(13)70143-8

[11] McGarrah RW, Crown SB, Zhang G-F, Shah SH, Newgard CB Cardiovascular metabolomics. Circ Res. 2018;122:1238–58. 29700070 10.1161/CIRCRESAHA.117.311002 PMC6029726

[12] Lotta LA, Scott RA, Sharp SJ, Burgess S, Luan J, Tillin T, Schmidt AF, Imamura F, Stewart ID, Perry JRB et al. Genetic predisposition to an impaired metabolism of the branched-chain amino acids and risk of type 2 diabetes: a Mendelian randomisation analysis. PLoS Med. 2016;13:1002179. 10.1371/journal.pmed.1002179 PMC5127513 27898682

[13] Wishart DS Metabolomics: applications to food science and nutrition research. Trends Food Sci Technol. 2008;19:482–93.

[14] Jones DP, Park Y, Ziegler TR Nutritional metabolomics: progress in addressing complexity in diet and health. Annu Rev Nutr. 2012;32:183–202. 22540256 10.1146/annurev-nutr-072610-145159 PMC4031100

[15] Floegel A, von Ruesten A, Drogan D, Schulze MB, Prehn C, Adamski J, Pischon T, Boeing H Variation of serum metabolites related to habitual diet: a targeted metabolomic approach in EPIC-Potsdam. Eur J Clin Nutr. 2013;67:1100–8. 23942179 10.1038/ejcn.2013.147

[16] Brennan L, Gibbons H, O'Gorman A An overview of the role of metabolomics in the identification of dietary biomarkers. Curr Nutr Rep. 2015;4:304–12.

[17] Bingham SA Biomarkers in nutritional epidemiology. Public Health Nutr. 2002;5:821–7. 12638591 10.1079/phn2002368

[18] Jenab M, Slimani N, Bictash M, Ferrari P, Bingham SA Biomarkers in nutritional epidemiology: applications, needs and new horizons. Hum Genet. 2009;125:507–25. 19357868 10.1007/s00439-009-0662-5

[19] Cooper AJM, Sharp SJ, Luben RN, Khaw K-T, Wareham NJ, Forouhi NG The association between a biomarker score for fruit and vegetable intake and incident type 2 diabetes: the EPIC-Norfolk study. Eur J Clin Nutr. 2015;69:449–54. 25387899 10.1038/ejcn.2014.246 PMC4704139

[20] Vázquez-Fresno R, Llorach R, Urpi-Sarda M, Lupianez-Barbero A, Estruch R, Corella D, Fitó M, Arós F, Ruiz-Canela M, Salas-Salvadó J et al. Metabolomic pattern analysis after Mediterranean diet intervention in a nondiabetic population: a 1- and 3-year follow-up in the PREDIMED study. J Proteome Res. 2015;14:531–40. 25353684 10.1021/pr5007894

[21] Guasch-Ferré M, Zheng Y, Ruiz-Canela M, Hruby A, Martínez-González MA, Clish CB, Corella D, Estruch R, Ros E, Fitó M et al. Plasma acylcarnitines and risk of cardiovascular disease: effect of Mediterranean diet interventions. Am J Clin Nutr. 2016;103:1408–16. 27099249 10.3945/ajcn.116.130492 PMC4881000

[22] Bondia-Pons I, Martinez JA, de la Iglesia R, Lopez-Legarrea P, Poutanen K, Hanhineva K, de los Ángeles Zulet M Effects of short- and long-term Mediterranean-based dietary treatment on plasma LC-QTOF/MS metabolic profiling of subjects with metabolic syndrome features: the Metabolic Syndrome Reduction in Navarra (RESMENA) randomized controlled trial. Mol Nutr Food Res. 2015;59:711–28. 25641909 10.1002/mnfr.201400309

[23] Toledo E, Wang DD, Ruiz-Canela M, Clish CB, Razquin C, Zheng Y, Guasch-Ferré M, Hruby A, Corella D, Gómez-Gracia E et al. Plasma lipidomic profiles and cardiovascular events in a randomized intervention trial with the Mediterranean diet. Am J Clin Nutr. 2017;106:973–83. 28814398 10.3945/ajcn.116.151159 PMC5611779

[24] MRC Epidemiology Unit, University of Cambridge. Fenland Study. [Internet] Cambridge (UK): MRC Epidemiology Unit; 2017; [cited 2017 July 8]. Available from: http://www.mrc-epid.cam.ac.uk/research/studies/fenland/.

[25] Bingham SA, Gill C, Welch A, Day K, Cassidy A, Khaw KT, Sneyd MJ, Key TJ, Roe L, Day NE et al. Comparison of dietary assessment methods in nutritional epidemiology: weighed records v. 24 h recalls, food-frequency questionnaires and estimated-diet records. Br J Nutr. 1994;72:619–43. 7986792 10.1079/bjn19940064

[26] Day N, McKeown N, Wong M, Welch A, Bingham S Epidemiological assessment of diet: a comparison of a 7-day diary with a food frequency questionnaire using urinary markers of nitrogen, potassium and sodium. Int J Epidemiol. 2001;30:309–17. 11369735 10.1093/ije/30.2.309

[27] Mulligan A, Luben RN, Bhaniani A, Parry-Smith DJ, O'Connor L, Khawaja AP, Forouhi NG, Khaw K-T A new tool for converting food frequency questionnaire data into nutrient and food group values: FETA research methods and availability. BMJ Open. 2014;4:e004503. 10.1136/bmjopen-2013-004503 PMC3975761 24674997

[28] Biocrates Life Sciences AG. AbsoluteIDQ p180 Kit. [Internet] Innsbruck (Austria): Biocrates Life Sciences AG; 2014; [cited 2016 July 12]. Available from: http://www.biocrates.com/products/research-products/absoluteidq-p180-kit.

[29] Illig T, Gieger C, Zhai G, Romisch-Margl W, Wang-Sattler R, Prehn C, Altmaier E, Kastenmuller G, Kato BS, Mewes HW et al. A genome-wide perspective of genetic variation in human metabolism. Nat Genet. 2010;42:137–41. 20037589 10.1038/ng.507 PMC3773904

[30] Siskos AP, Jain P, Römisch-Margl W, Bennett M, Achaintre D, Asad Y, Marney L, Richardson L, Koulman A, Griffin JL et al. Interlaboratory reproducibility of a targeted metabolomics platform for analysis of human serum and plasma. Anal Chem. 2017;89:656–65. 27959516 10.1021/acs.analchem.6b02930 PMC6317696

[31] De Lucia Rolfe E, Loos RJF, Druet C, Stolk RP, Ekelund U, Griffin SJ, Forouhi NG, Wareham NJ, Ong KK Association between birth weight and visceral fat in adults. Am J Clin Nutr. 2010;92:347–52. 20519560 10.3945/ajcn.2010.29247

[32] Brage S, Brage N, Franks PW, Ekelund U, Wareham NJ Reliability and validity of the combined heart rate and movement sensor Actiheart. Eur J Clin Nutr. 2005;59:561–70. 15714212 10.1038/sj.ejcn.1602118

[33] Friedewald WT, Levy RI, Fredrickson DS Estimation of the concentration of low-density lipoprotein cholesterol in plasma, without use of the preparative ultracentrifuge. Clin Chem. 1972;18:499–502. 4337382

[34] Bonora E, Formentini G, Calcaterra F, Lombardi S, Marini F, Zenari L, Saggiani F, Poli M, Perbellini S, Raffaelli A et al. HOMA-estimated insulin resistance is an independent predictor of cardiovascular disease in type 2 diabetic subjects: prospective data from the Verona Diabetes Complications Study. Diabetes Care. 2002;25:1135–41. 12087010 10.2337/diacare.25.7.1135

[35] White IR, Royston P, Wood AM Multiple imputation using chained equations: issues and guidance for practice. Stat Med. 2011;30:377–99. 21225900 10.1002/sim.4067

[36] Benjamini Y, Yekutieli D The control of the false discovery rate in multiple testing under dependency. Ann Statist. 2001;29:1165–88.

[37] Tzoulaki I, Ebbels TMD, Valdes A, Elliott P, Ioannidis JPA Design and analysis of metabolomics studies in epidemiologic research: a primer on -omic technologies. Am J Epidemiol. 2014;180:129–39. 24966222 10.1093/aje/kwu143

[38] Harrell FE Jr, Lee KL, Mark DB Multivariable prognostic models: issues in developing models, evaluating assumptions and adequacy, and measuring and reducing errors. Stat Med. 1996;15:361–87. 8668867 10.1002/(SICI)1097-0258(19960229)15:4<361::AID-SIM168>3.0.CO;2-4

[39] Stringhini S, Tabak AG, Akbaraly TN, Sabia S, Shipley MJ, Marmot MG, Brunner EJ, Batty GD, Bovet P, Kivimäki M Contribution of modifiable risk factors to social inequalities in type 2 diabetes: prospective Whitehall II cohort study. BMJ. 2012;345:e5452. 22915665 10.1136/bmj.e5452 PMC3424226

[40] Richiardi L, Bellocco R, Zugna D Mediation analysis in epidemiology: methods, interpretation and bias. Int J Epidemiol. 2013;42:1511–9. 24019424 10.1093/ije/dyt127

[41] Schooneman MG, Vaz FM, Houten SM, Soeters MR Acylcarnitines: reflecting or inflicting insulin resistance?. Diabetes. 2013;62:1–8. 23258903 10.2337/db12-0466 PMC3526046

[42] Shah SH, Sun J-L, Stevens RD, Bain JR, Muehlbauer MJ, Pieper KS, Haynes C, Hauser ER, Kraus WE, Granger CB et al. Baseline metabolomic profiles predict cardiovascular events in patients at risk for coronary artery disease. Am Heart J. 2012;163:844–50..e1. 22607863 10.1016/j.ahj.2012.02.005

[43] Rizza S, Copetti M, Rossi C, Cianfarani MA, Zucchelli M, Luzi A, Pecchioli C, Porzio O, Di Cola G, Urbani A et al. Metabolomics signature improves the prediction of cardiovascular events in elderly subjects. Atherosclerosis. 2014;232:260–4. 24468136 10.1016/j.atherosclerosis.2013.10.029

[44] Li Z, Vance DE Phosphatidylcholine and choline homeostasis. J Lipid Res. 2008;49:1187–94. 18204095 10.1194/jlr.R700019-JLR200

[45] Soininen P, Kangas AJ, Würtz P, Suna T, Ala-Korpela M Quantitative serum nuclear magnetic resonance metabolomics in cardiovascular epidemiology and genetics. Circ Cardiovasc Genet. 2015;8:192–206. 25691689 10.1161/CIRCGENETICS.114.000216

[46] Marshall DL, Criscuolo A, Young RSE, Poad BLJ, Zeller M, Reid GE, Mitchell TW, Blanksby SJ Mapping unsaturation in human plasma lipids by data-independent ozone-induced dissociation. J Am Soc Mass Spectrom. 2019;30(9):1621–30. 31222675 10.1007/s13361-019-02261-z

[47] Martínez-González MÁ, Hershey MS, Zazpe I, Trichopoulou A Transferability of the Mediterranean diet to non-Mediterranean countries. What is and what is not the Mediterranean diet. Nutrients. 2017;9(11):1226. 10.3390/nu9111226 PMC5707698 29117146

[48] da Silva R, Bach-Faig A, Raidó Quintana B, Buckland G, Vaz de Almeida MD, Serra-Majem L Worldwide variation of adherence to the Mediterranean diet, in 1961–1965 and 2000–2003. Public Health Nutr. 2009;12:1676–84. 19689839 10.1017/S1368980009990541

