# Supplementary material for: A Combination of Metabolites Predicts Adherence to the Mediterranean Diet Pattern and Its Associations with Insulin Sensitivity and Lipid Homeostasis in the General Population: The Fenland Study, United Kingdom
Source: J Nutr. 2019 Oct 26;150(3):568–78. doi: 10.1093/jn/nxz263 (PMC7315099; doi:10.1093/jn/nxz263)
Supplement: nxz263_Supplementary_Files [file nxz263_supplementary_files.zip › Tong_JN_2020_SupplementaryMaterials.docx]

**Online Supplementary Material

A combination of metabolites predicts adherence to the Mediterranean diet pattern and its associations with insulin sensitivity and lipid homeostasis in the general population: the Fenland Study, United Kingdom**
Authors: TYN Tong, Albert Koulman, Julian L. Griffin, Nicholas J. Wareham, Nita G. Forouhi, Fumiaki Imamura

**Supplemental Text.**

*Blood sampling and assay details of targeted metabolomics and the other biochemical measures*

The assay details were described previously as well (supplemental references 1,2). Fasting venous blood samples were taken at a clinical visit of the study. Blood samples were placed on ice, centrifuged for plasma aliquots, and stored at -80°C. The metabolite concentrations in the plasma were measured in the Fenland study by the AbsoluteIDQ® Biocrates p180 Kit (Biocrates Life Sciences AG, Innsbruck, Austria) (supplemental references 3,4). We used a Waters Acquity ultra-performance liquid chromatography (UPLC; Waters ltd, Manchester, UK) system coupled to an ABSciex 5500 Qtrap mass spectrometer (Sciex ltd, Warrington, UK). Samples were derivatised and extracted using a Hamilton STAR liquid handling station (Hamilton Robotics Ltd, Birmingham, UK). Flow injection analysis coupled with tandem mass spectrometry (FIA-MS/MS) using multiple reaction monitoring (MRM) in positive mode ionisation was performed to measure the relative levels of acylcarnitines, phosphatidylcholines, lysophosphatidylcholines and sphingolipids. The level of hexose was measured in negative ionisation mode. Ultra-performance liquid chromatography coupled with tandem mass spectrometry (UPLC-MS/MS) using MRM was performed to measure the concentration of amino acids and biogenic amines. The chromatography consisted of a 5 minute gradient starting at 100% aqueous (0.2% Formic acid) increasing to 95% acetonitrile (0.2% Formic acid) over a Waters Acquity UPLC BEH C18 column (2.1 x 50 mm, 1.7 μm, with guard column). Isotopically labelled internal standards are integrated within the Biocrates p180 Kit for quantification.

Data were processed in the Biocrates MetIDQ software. Raw metabolite readings underwent extensive quality control procedures. Firstly, we excluded from any further analysis metabolites for which the number of measurements below the limit of quantitification (LOQ) exceeded 5% of measured samples. Excluded metabolites were asymmetric dimethylarginine, carnosine, dihydroxyphenylalanine, dopamine, nitro-tyrosine, putrescine, spermine, three acylcarnitines (OH-c4, OH-c6, and c6), phosphatidylcholine diacyl c30:2, and four sphingomyelins (c24:1, c22:3, c22:0, and c26:1). Secondly, in samples with detectable but not quantifiable peaks, we assigned random values between 0 and the run-specific LOQ of a given metabolite. Finally, we corrected for batch-effects with a “location-scale” approach, i.e. with normalization for mean and standard deviation of batches (supplemental reference 5).

Plasma glucose was measured using the hexokinase method with a lower limit of detection of 0.5 mmol/L and interassay CV of 1.8%. Plasma triglyceride and HDL cholesterol were measured using standard enzymatic methods; interassay CVs were 4.8% and 3.9% respectively. Insulin was determined using a two-step time resolved flourometric assay with a lower limit of detection of 1.3 pmol/L. Plasma vitamin C was measured from blood samples taken into heparin tubes and centrifuged (10 minutes) within 30 minutes of collection. Plasma was aliquoted into cryovials with a standardised volume of metaphosphoric acid (10%) and stored at -80°C. Plasma vitamin C concentration was measured by fluorometric assay within 2 months, with a lower limit of detection of 10.0 µmol/L. Between batch imprecision was 7.9% at 27.1 µmol/L and 5.0% at 89.7 µmol/L.

# Supplemental Table 1: Mediterranean diet score scoring criteria.

| **Component** | **Recommended intake***^1^* | **Score of 0***^1^* | **Score of 1***^1^* |
| --- | --- | --- | --- |
| Vegetables*^2^* | ≥6/d | 0/d | ≥6/d |
| Legumes*^2^* | ≥2/wk | 0/wk | ≥2/wk |
| Fruits*^3^* | 3-6/d | 0/d | 3-6/d |
| Nuts*^3^* | 1-2/d | 0/d | 1-2/d |
| Cereals*^3^* | 3-6/d | 0/d | 3-6/d |
| Dairy*^3^* | 2/d | 0/d | 1.5-2.5/d |
| Fish*^2^* | ≥2/wk | 0/wk | ≥2/wk |
| Red meat*^5^* | ˂2/wk | ≥4/wk | ˂2/wk |
| Processed meat*^5^* | ≤1/wk | ≥2/wk | ≤1/wk |
| White meat*^3^* | 2/wk | 0/wk | 1.5-2.5/wk |
| Egg*^3^* | 2-4/wk | 0/wk | 2-4/wk |
| Potato*^5^* | ≤3/wk | ≥6/wk | ≤3/wk |
| Sweets*^5^* | ≤2/wk | ≥4/wk | ≤2/wk |
| Alcohol*^4^* | 2/d for men,  1/d for women | ≥4/d for men,  ≥2/d for women | 1.5-2.5/d for men,  0.5-1.5/d for women |
| Olive oil*^6^* | Principal source of  dietary lipids | Non-consumers | Consumers |

*^1^* All recommendations are in number of servings per day or per week and we used continuous scoring for all components except olive oil.
*^2^* For these components for which a high consumption was recommended, continuous scores from 0 to 1 were assigned proportionally from no consumption to meeting the recommended level of consumption.
*^3^* For components for which moderate consumption was recommended, we assigned a score of 1 for consumption within the recommendation levels and 0 for no consumption, with consumption levels in between scored proportionately. Overconsumption (double the mid-point value of the recommended intake) was penalised and received a maximum score of 0.5, with consumption between the recommended level and the penalty point scored proportionally.
*^4^* For alcohol we assigned a score of 1 for consumption levels within recommendation. Non-consumption was scored 0.5 while overconsumption was scored 0.
*^5^* For these components for which a low consumption was recommended, consumption below the recommended levels was assigned a score of 1 and double the recommended levels were assigned a score of 0, with levels in between scored proportionally.
*^6^* For olive oil all non-consumers were scored 0 and all consumers 1.


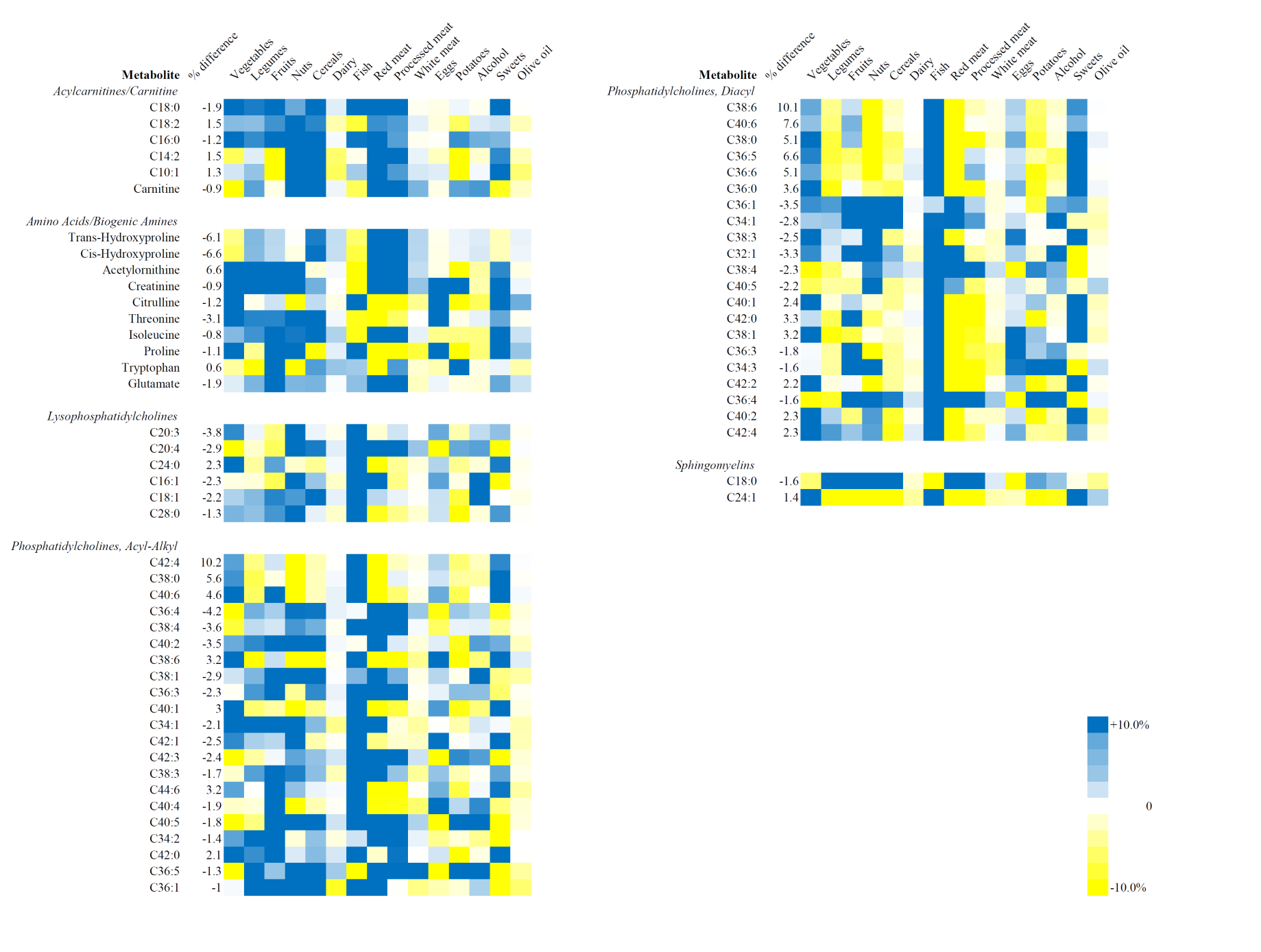


**Supplemental Figure 1. Contribution of dietary components to the association between the Mediterranean diet and metabolite levels: the Fenland Study.** % difference in metabolite levels based on linear association between per standard deviation of Mediterranean diet score and per log unit of metabolites. Contribution of each dietary component assessed based on linear regression between per standard deviation of Mediterranean diet score and per log unit of metabolites, additionally adjusted for the dietary component of interest. Blue indicates contribution of the dietary component, while yellow indicates no contribution. For acylcarnitines, phospholipids, and sphingolipids, number of carbons and double bonds of acyl moieties are presented.

**Supplemental Table 2.** Associations of the Mediterranean diet with blood pressure and 2 hour glucose and estimated contributions to metabolites to the associations: the Fenland Study.

|  | **Systolic blood pressure,**  **mm Hg** | | **Diastolic blood pressure,**  **mm Hg** | | **LDL-C,  mmol/L** | | **2 hour glucose, mmol/L** | |
| --- | --- | --- | --- | --- | --- | --- | --- | --- |
|  | Difference  (95% CI)*^1^* | % Attenuation*^2^* | Difference  (95% CI)*^1^* | % Attenuation*^2^* | Difference  (95% CI)*^1^* | % Attenuation*^2^* | Difference  (95% CI)*^1^* | % Attenuation*^2^* |
| Reference Model*^2^* | -0.12 (-0.41,0.17) |  | -0.09 (-0.28,0.10) |  | -0.01 (-0.03, 0.01) |  | -0.03 (-0.07,0.00) |  |
| Adjusted for metabolite sub-classes | | | | | | | | |
| + acylcarnitines | -0.06 (-0.35, 0.23) | 51.0 | -0.04 (-0.23, 0.16) | 51.6 | 0.01 (-0.01, 0.03) | 202.9 | -0.03 (-0.06, 0.01) | 19.5 |
| + amino acids /biogenic amines | -0.17 (-0.50, 0.16) | -5.6 | -0.08 (-0.30, 0.14) | 12.8 | -0.01 (-0.04, 0.00) | -31,1 | -0.03 (-0.07, 0.01) | 12.9 |
| + lysophosphatidylcholines | 0.01 (-0.28, 0.31) | 110.9 | -0.02 (-0.21, 0.18) | 77.5 | 0.00 (-0.02, 0.02) | 130.7 | -0.03 (-0.07, 0.00) | 6.3 |
| + phosphatidylcholine acyl-alkyls | -0.06 (-0.38, 0.27) | 70.7 | -0.04 (-0.24, 0.17) | 71.2 | -0.01 (-0.03, 0.01) | 7.1 | -0.02 (-0.06, 0.02) | 38.5 |
| + phosphatidylcholine diacyls | -0.02 (-0.34, 0.31) | 87.1 | 0.03 (-0.19, 0.25), | 155.3 | -0.02 (-0.04, -0.00) | -15.6 | -0.03 (-0.07, 0.01) | 18.7 |
| + sphingolipids | -0.11 (-0.41, 0.19) | 14.8 | -0.07 (-0.26, 0.12) | 24.0 | 0.00 (-0.02, 0.02) | 104 | -0.02 (-0.06, 0.01) | 26.1 |
| Adjustment for the metabolite score | | | | | | | | |
| + metabolite score*^3^* | -0.18 (-0.55, 0.19) | 20.2 | -0.05 (-0.30, 0.20) | 52.6 | -0.00 (-0.03, 0.02) | 81.6 | -0.03 (-0.07, 0.01) | 33.6 |

^1^ Values shown are unit differences in cardiovascular risk factors, based on β coefficients from linear regression fitted to data from 10,806 adults recruited in 2005-2015 in the Fenland Study. All estimates were adjusted for age, sex, test site, education level, income, occupation, medication use, family history of diabetes, objectively measured physical activity, smoking, BMI and waist circumference.

^2^ Percent changes in β coefficients were calculated as changes in β coefficients from those of ‘Reference model’ upon statistical adjustment for the metabolite score or metabolite sub-classes (mediation analysis).

^3^ Metabolite score included all 66 metabolites associated with the Mediterranean diet derived in a random half of the total dataset and validated in the second half, weighted by their respective regression coefficients.

**Supplemental references**

1 Lotta LA, Scott RA, Sharp SJ, Burgess S, Luan J, Tillin T, Schmidt AF, Imamura F, Stewart ID, Perry JRB, *et al.* Genetic Predisposition to an Impaired Metabolism of the Branched-Chain Amino Acids and Risk of Type 2 Diabetes: A Mendelian Randomisation Analysis. *PLoS Med* 2016;**13**:1–22.

2 O’Connor L, Imamura F, Brage S, Griffin SJ, Wareham NJ, Forouhi NG. Intakes and sources of dietary sugars and their association with metabolic and inflammatory markers. *Clin Nutr* 2018;**37**:1313–22.

3 Illig T, Gieger C, Zhai G, Romisch-Margl W, Wang-Sattler R, Prehn C, Altmaier E, Kastenmuller G, Kato BS, Mewes HW, *et al.* A genome-wide perspective of genetic variation in human metabolism. *Nat Genet* 2010;**42**:137–41.

4 Walsh BH, Broadhurst DI, Mandal R, Wishart DS, Boylan GB, Kenny LC, Murray DM. The metabolomic profile of umbilical cord blood in neonatal hypoxic ischaemic encephalopathy. *PLoS One* 2012;**7**:e50520.

5 Johnson WE, Li C, Rabinovic A. Adjusting batch effects in microarray expression data using empirical Bayes methods. *Biostatistics* 2007;**8**:118–27.
